# Comparison of Push-out Bond Strength of Gutta-percha to Root Canal Dentin in Single-cone and Cold Lateral Compaction Techniques with AH Plus Sealer in Mandibular Premolars

**DOI:** 10.15171/joddd.2015.040

**Published:** 2015-12-30

**Authors:** Hadi Mokhtari, Saeed Rahimi, Mohammad Forough Reyhani, Saeedeh Galledar, Hamid Reza Mokhtari Zonouzi

**Affiliations:** ^1^Dental and Periodontal Research Center, Tabriz University of Medical Sciences, Tabriz, Iran; ^2^Assistant Professor, Department of Endodontics, Faculty of Dentistry, Tabriz University of Medical Sciences, Tabriz, Iran; ^3^Professor, Department of Endodontics, Faculty of Dentistry, Tabriz University of Medical Sciences, Tabriz, Iran; ^4^Assosiate Professor, Department of Endodontics, Faculty of Dentistry, Tabriz University of Medical Sciences, Tabriz, Iran; ^5^Post-graduate Student, Department of Endodontics, Faculty of Dentistry, Tabriz University of Medical Sciences, Tabriz, Iran; ^6^Assistant Professor, Department of Endodontics, Faculty of Dentistry, Tabriz University of Medical Sciences, Tabriz, Iran

**Keywords:** Push-out bond strength, gutta-percha, root dentin

## Abstract

***Background and aims.*** The single-cone technique has gained some popularity in some European countries. The aim of the present study was to compare the push-out bond strength of gutta-percha to root canal dentin with the single-cone and cold lateral compaction canal obturation techniques.

***Materials and methods***. The root canals of 58 human mandibular premolars were prepared using modified crown-down technique with ProTaper rotary files up to #F3as a master apical file (MAF) and divided randomly into groups A and B based on canal obturation technique. In group A (n = 29) the root canals were obturated with single-cone technique with #F3(30/.09) ProTaper gutta-percha, which was matched with MAF in relation to diameter, taper and manufacturer; in group B (n = 29) the canals were obturated with gutta-percha using cold lateral compaction technique. In both groups AH plus sealer were used. After two weeks of incubation, three 2-mm slices were prepared at a distance of 2 mm from the coronal surface and push-out test was carried out. Data were analyzed with descriptive statistics using independent samples t-test.

***Results.*** There were statistically significant differences between two groups. The mean push-out bond strength was higher in group B (lateral compaction technique) compared to group A (single-cone technique; P < 0.05).

***Conclusion***. Use of single-cone technique for obturation of root canals resulted in a lower bond strength compared to cold lateral compaction technique.

## Introduction

The aims of endodontic treatment are cleaning, shaping and obturation of the root canal system.^1^ The root canal system should be sealed apically, coronally, and laterally. Maintenance of adequate obturation is of critical importance for prevention of bacterial microleakage.^2^ Different obturation techniques are used to achieve maximum adaptation of filling materials with the root canal space; these techniques consist of thermoplastic, cold lateral compaction and single-cone techniques.^3^ With the advent of nickel-titanium (Ni-Ti) systems, preparation of root canals with rotary system has become very popular. Use of rotary instruments, with crown-down technique, results in easy and fast preparation of root canals, decreasing procedural errors and preserving the natural curve of the root canal.^4-8^

The most commonly used technique for root canal obturation is the cold lateral compaction technique;^9^ however, this technique is difficult and time-consuming and might result in homogeneity defect of gutta-percha mass, a high percentage of sealer in the apical portion of the canal, poor adaptation of gutta-percha with canal walls and root fracture.^10^ To overcome these problems, the single-cone technique has become very popular; this technique uses gutta-percha cones which are matched with rotary instruments in relation to diameter, taper and manufacturer. It is a very easy and fast technique and can produce a homogenous mass of gutta-percha without air bubbles and sealers between gutta-percha cones.^11,12^ An important characteristic of this technique is the dimensional stability of gutta-percha, allowing packing of maximum amount of gutta-percha cones in the root canal and minimizing the amount of sealer in the root canal space.^13-15^ Ideal adhesion of root cannel filling material to the root dentin is one of the main criteria to evaluate the clinical efficacy of obturation techniques, and one of the techniques used to evaluate the adhesion is the push-out bond strength test, which is supposed to create conditions similar to clinical conditions.^16^ Some in vitro studies using a leakage technique with fluid movement did not find a difference after root filling of oval-shaped canals with single cone and lateral compaction technique.^17^ Also the lateral compaction technique did not differ from the single cone technique with respect to the radiographic quality of the root filling in some studies.^18^ However, little studies to date have evaluated the bond strength of single-cone technique with the use of matching gutta-percha. Therefore, the aim of the present study was to compare the push-out bond strength of gutta-percha to root canal dentin with the use of single-cone and cold lateral compaction techniques with AH Plus sealer.

## Materials and Methods

In the present study, 58 one-rooted mandibular premolars were selected based on these criteria: 1) One-rooted and single canal teeth with similar apical foramen size; teeth with apical constriction larger than #20 K-file were excluded; 2) Straight and mature roots; 3) Absence of apical resorption; 4) No history of endodontic treatment.

The selected teeth were cleaned of any soft tissue remnants and stored in 0.5% chloramine T solutions (Merck KGaA, Darmstadt, Germany). Then the tooth crowns were removed to achieve a standard root length of 14 mm. A #10 K-file (Mani Inc., Tochigi, Japan) was placed in each root canal to determine the root length by observing the file tip at the apical foramen. Then the working length (WL) was determined 1 mm short of the file penetration length. The root canals were prepared by crown-down technique using Pro Taper rotary instruments (Dentsply-Maillefer, Ballaigues, Switzerland). Root canal preparation was carried out by shaping Sx and S_1_ files in the cervical third, by S_2_ file in the middle third and by F_1_, F_2_ and F_3_ finishing files up to the entire working length. Each instrument set was used for preparation of 5 root canals and the root canals were irrigated with 5 mL of 2.5% NaOCl between instruments. After completion of root canal shaping and cleaning, the canals were irrigated with 5 mL 17% EDTA solution for 1 minute, followed by 10 mL of saline solution. Then the root canals were dried with #30 paper points and prepared for obturation.

Based on the root canal obturation technique, the samples were randomly divided into two groups of A and B (n = 29). In group A, single-cone technique was used for canal obturation. F_3_ (30/.09) Pro Taper gutta-percha cone (Dentsply-Maillefer, Ballaigues, Switzerland) which was matched with the MAF in relation to diameter, taper and manufacturer, was coated with AH plus sealer (Dentrey Dentsply, Germany) as master cone and placed in the root canal space up to the WL. In group B, the root canals were obturated with lateral compaction technique; #30/.02 gutta-percha cone was coated with AH plus sealer, as master cone, and placed in the root canal up to the entire WL. Then #20/.02 accessory cones were placed in the root canals. A #25 Ni-Ti finger spreader (Mani Inc., Tochigi, Japan) was used to condense gutta-percha. The quality of root canal obturation was evaluated by periapical radiography. Then a hot instrument was used to remove extra gutta-percha from canal orifices. The canal orifices were sealed with a Coltosol (Coltene Whaledent, Mahwah, NJ, USA) as a temporary restorative material. The samples underwent a push-out test after a 2-week incubation period at 37°C and 100% relative humidity.

### Push-out Test 

The push-out test was carried out in a uniform manner in both groups. In each tooth five 2-mm-thick dentin disks were prepared using a diamond saw (SP1600 Microtome; Leica, Nußloch, Germany) perpendicular to the long axis of the root under water cooling. The coronal and apical disks were evaluated in relation to the centrality of the root canal. Only those disks were selected for the push-out tests that exhibited canal centrality, a homogeneous sealer layer and no air bubbles at least three disks for each tooth. In the dentin disks selected for the push-out test the coronal and apical radii were measured using a digital caliper with +/−0.02 mm accuracy (Mitutoyo absolute 573-281, America) and the apical area was marked with a special marker. Then the filling material underwent a dislodging force in the apico-coronal direction at a crosshead speed of 0.5 mm/min using the stainless steel plunger, measuring 0.8 mm in diameter, in a universal testing machine (Hounsfield Test Equipment, Model H5K-s, Surrey, England). The maximum force that dislodged the material was measured in Newton and converted to MPa using the formula below:^19^

$$\left(MPa\right)\;=\frac{\text{Maximum load (N)}}{\text{Adhesion area (mm2)}}$$

The adhesion area was calculated using the following formula: π (R+r) [h^2^ +(R-r)2]^0.5^

In which π = 3.14, R is the coronal radius, r is the apical radius and h is the slice thickness.

Data were analyzed with descriptive statistics (frequencies, means and standard deviations) using independent samples t-test. Statistical significance was set at P < 0.05.

## Results

Table 1 presents the means and standard deviations of push-out bond strength values with the two canal obturation techniques. The results of Kolmogorov-Smirnov test showed normal distribution of bond strength values and data were parametric (Kolmogorov-Smirnov z = 1.05, P = 0.22). Independent two-sample t-test was used to compare mean bond strength values between the two groups. The results showed significant differences in mean bond strength values between the two groups (P = 0.008). It was concluded at a 95% confidence interval that the mean push-out bond strength values in group B (lateral compaction technique) were higher than those in group A (single-cone technique). The results of this test are presented in Table 2.

**Table 1:** The means and standard deviations of push-out bond strength values in the two study groups

**Groups**	**Mean ± SD**	**Minimum**	**Maximum**
**Group B (lateral compaction technique)**	4.03 ± 2.51	0.669	11.328
**Group A (single cone technique)**	2.36 ± 0.79	4.027	1.51

**Table 2:** The results of two-sample t-test

	**F**	**P**	**Degree of freedom**	**t**	**P**
**Variances are equal**	7.43	0.01	33	2.559	0.015
**Variances are unequal**			23.807	2.888	0.008

## Discussion

Resistance of the root canal filling material against dislodgment from the root canal dentin is effective in preserving the integrity of the sealer‒dentin interface during tooth flexure and post space preparation,^20^and the push-out test is a reliable technique to measure bond strength between the root canal obturation and root dentin.^21^ In the present study, the push-out bond strength values with both single-cone and lateral compaction techniques were higher than these in similar studies such as studies by Fisher and Cecchin,^22,23^ which might be attributed to the formation of a covalent bond with the epoxide open ring in collagen amino groups, low polymerization shrinkage and the long-term dimensional stability of this sealer.^24,25^

Based on the results of the present study, the single-cone technique exhibited a lower bond strength compared to the cold lateral compaction technique, contrary to the results reported by Nagas et al,^10^ who showed that use of single-cone technique with a master cone with 0.06 taper might result in an increase in bond strength of the filling material to dentin compared to the lateral compaction technique. This discrepancy might be explained by differences in methodologies between the two studies. In that study one-rooted anterior maxillary teeth were used and during the push-out test samples with ovoid canal shapes were excluded from the study; however, the present study was carried out on mandibular premolars, which have a high rate of ovoid and ribbon-shaped canals based on morphologic studies.^26,27^ Previous studies have shown that use of the single-cone technique to obturate root canals with an irregular shape results in formation of bubbles,^28,29^ and an increase in the volume of sealer in the root canal filling material.^30^ The main disadvantage of the single-cone technique is poor adaptation of the single master cone with the coronal and middle thirds of irregularly shaped root canals, which increases the dispersion of sealer and leakage of liquids.^31^All these factors can justify the low bond strength with the single-cone technique in the present study. Therefore, it is recommended that single-cone technique be used only in round root canals with a circular preparation and if such a technique is used with ovoid canals it is advisable to place several cones other than the main cone without compaction with a finger spreader in the canal to increase the relative percentage of gutta-percha in the coronal region of the filling.^32^

In the present study, the bond strength of single cone technique is compared with cold lateral compaction technique in mandibular premolars. Further research is required to comparison of the bond strength of single cone technique with various obturation techniques according to root canal form.

## Conclusion

On the whole, considering the limitations of *in vitro* studies, the results of this study showed that use of the single-cone technique, with a master cone matching the root canals prepared with tapered rotary instruments, resulted in a lower bond strength compared to the use of cold lateral compaction technique. However, future studies are necessary in this respect.

## Acknowledgments

This research was carried out by financial support of the Vice Chancellor for Research at Tabriz University of Medical Sciences. The authors declare that they have no competing interests.
